# Quantifying *Microstegium vimineum* Seed Movement by Non-Riparian Water Dispersal Using an Ultraviolet-Marking Based Recapture Method

**DOI:** 10.1371/journal.pone.0063811

**Published:** 2013-09-12

**Authors:** Daniel R. Tekiela, Jacob N. Barney

**Affiliations:** Department of Plant Pathology, Physiology, and Weed Science, Virginia Tech, Blacksburg, Virginia, United States of America; University of Konstanz, Germany

## Abstract

*Microstegium vimineum* is a shade tolerant annual C_4_ invasive grass in the Eastern US, which has been shown to negatively impact species diversity and succession in hardwood forests. To date, empirical studies have shown that population expansion is limited to <1 m yr^−1^, which is largely driven by gravity dispersal. However, this likely does not fully account for all mechanisms of population-scale dispersal as we observe greater rates of population expansion. Though water, both riparian and non-riparian water (i.e., ephemeral overland flow), have been speculated mechanisms for *M. vimineum* dispersal, few studies have empirically tested this hypothesis. We designed an experiment along the slopes of a Southwest Virginia hardwood forest to test the role of non-riparian water on local seed dispersal. We developed a seed marking technique by coating each seed with an ultraviolet (UV) powder that did not affect buoyancy to aid *in situ* seed recapture. Additionally, a new image analysis protocol was developed to automate seed identification from UV photos. Total seed mobility (summation of individual seed movement within each transect) was positively correlated with precipitation. Over a period of one month with 52.32 mm of precipitation, the maximum dispersal distance of any single recaptured seed was 2.4 m, and the average distance of dispersed seed was 0.21±0.04 m. This is the first quantitative evidence of non-riparian water dispersal in a forest understory, which accounts for an additional pathway of population expansion.

## Introduction

Because the sessile nature of the plant kingdom, the dispersal of viable propagules is crucial in determining the spatial distribution, demographics, and spread of nearly all plant species [1]. In the context of invasive species, spatial spread is a defining character: invasiveness is “produc[ing] reproductive offspring in areas distant from sites of introduction (approximate scales: >100 m over <50 years for taxa spreading by seeds and other propagules; >6 m/3 years for taxa spreading by roots, rhizomes, stolons or creeping stems)” [2]. Though this definition is related to long distance dispersal, it shows how critical effective dispersal is to the success of invasive species. Population expansion is the result of numerous local dispersal events that are important to characterize to understand demographic rates, as well as design effective management strategies [3]. In fact, local population expansion has been used to characterize the “aggressiveness” of a clonal invasive plant, demonstrating an additional utility of understanding population spread [4]. Therefore, our aim was to explore one poorly understood mechanism of population expansion in *Microstegium vimineum*, one of the worst invasive species of the Eastern United States.

*Microstegium vimineum* (Trin.) A. Camus (Japanese stiltgrass, Nepalese browntop, Chinese packing grass) is an annual C_4_ invasive grass, which has quickly spread across the Eastern US since its initial introduction in 1919 into Tennessee [5]. A 2008 survey reported *M. vimineum* on 260,000 ha in the Southeast, and eight state and federal agencies have listed *M. vimineum* among their greatest ongoing or potential management problems, behind only kudzu (*Pueraria montana* var. *lobata*) and multiflora rose (*Rosa multiflora*) [6]. Despite being a C_4_ grass, *M. vimineum* is capable of growing vigorously in low light conditions of 25–50% of full sun [7], allowing it to colonize the shaded hardwood forests of the Northeast, Mid-Atlantic and Southeastern US. *Microstegium vimineum* is most commonly found in dense monocultures along forest edges and trails [8], [9], but often occurs in lower densities in the shaded forest interior [10] where it can reproduce in as little as 5% ambient light [11]. Even in these extreme low light environments, *M. vimineum* is capable of reducing biodiversity of native plant communities [12], and importantly, it impacts forest successional patterns through the suppression of tree seedling recruitment [13]–[15].

Like many invasive species, human activity is thought to disperse *M. vimineum* long distances through soil movement activities [16], but it does not appear to have any clear adaptations to assist in seed dispersal, suggesting that stem lodging and gravity are the primary mechanisms of local dispersal [17]. However, current reports of local dispersal ranging from 0.25 to 1 m yr^−1^ [18]–[21] do not explain how *M. vimineum* has expanded to cover over 260,000 ha in <100 years. This has lead to common speculation of water-based seed movement to fill the gap between observed *in situ* rapid population expansion and empirical data of seed movement.

Though the seed (∼3 mm in length) has no clear adaptations for dispersal, there are anecdotal reports of *M. vimineum* being dispersed by water. These reports refer to rivers acting as corridors [16], [22], [23], or flooding events [10], [16], [18], [24], but surprisingly, little empirical evidence exists of *M. vimineum* water dispersal. Romanello [25] showed that *M. vimineum* is capable of being dispersed through channelized water via drainage pipe discharge beneath roads, but only focused on the presence of seed, not the quantity or distance. Warren et al. [26] relocated seed and found seed near channelized water moved significantly further than that on dry land, suggesting water was the mechanism of dispersal.

We hypothesize that seed is being moved via ephemeral non-riparian water dispersal (i.e., dispersal by means of water not associated with streams, rivers, or commonly channeled waterways). Evidence for non-riparian water dispersal is likely limited [27] because locating small, dispersed seed is extremely difficult in the environment [28], [29]. Tracking (i.e., Lagrangian method) individual seeds requires the ability to follow seeds in real time for potentially long distances. This requires an extraordinary amount of time and effort for each individual seed, limiting the amount of seed that can be tracked. For this reason, trapping (i.e., Eulerian method) propagules arriving at a specific location is most often used because it is easier to implement [28]. Though trapping is simpler, it is difficult to know the source of each trapped seed, leading to conspecific contamination from unintended seed sources. Also, the location of traps is critical to correctly assessing seed dispersal [30], therefore it requires predicting a species dispersal kernel, which is impossible for a species that has not already been studied. We avoid these difficulties by implementing a unique trapping method that is able to differentiate between the seed population of interest and conspecific populations, which we will refer to as a recapture method.

Because we are only interested in how rain events may disperse *M. vimineum* seed locally (i.e., at the population level) the area in which the seed is dispersed is relatively small, making it possible to scan the entire potential dispersal area. By knowing the entire area of potential seed dispersal, we can avoid the limitations of trapping and tracking and instead use a recapture method [31]. Recapture requires that seeds be identifiable *in situ* from a point source introduction, thus we label the seeds to assist in recapture. This method is similar to the mark-recapture methodology common in ethology [32]. Because we choose to relocate the seed itself instead of germinated seedlings, we avoid the potential confounding environmental influence factors when using “realized dispersal” (i.e., dispersal and germination) data like previous *M. vimineum* dispersal studies (however, see Warren et al. [24]).

Thus, our objectives were to determine the following: 1) the efficacy of seed identification of our recapture method; 2) if *M. vimineum* is dispersed by non-riparian water dispersal; and 3) how precipitation affects dispersal distance.

## Methods

### Seed marker testing

This study required marking *M. vimineum* seed that easily distinguished marked from unmarked seed *in situ*, would not effect buoyancy, would remain on the seed following multiple rain events, and was easily repeatable. It is important that the marking method not impact seed buoyancy, as buoyancy is a major component of water dispersal and is important for many hydrochorous plant species [33], [34]. We tested standard orange marking paint (Rust-oleum Precision Line), and the ultraviolet powders UVSWR, UVXPBR, and UVLWR (LDP, LLC). The marking paint was sprayed to lightly and entirely coat each seed, and the UV powders were placed in bottles with seeds and agitated to coat the entire seed. The UV-coated seeds were sieved to remove any additional powder residue that did not adhere to the seed. All powder and paint coated the seed well except for UVLWR. To test for water fastness we agitated coated seed in a bottle of tap water three times, dried and checked for UV dye integrity. All treatments remained on the seed through the disturbance except UVLWR, so it was eliminated from further consideration.

To examine the impact of the markers on seed buoyancy, we placed 5 replications of 10 seeds for each of the remaining three treatments, and an unmarked control treatment in 5 cm diameter containers filled with deionized water. The containers were left undisturbed and the number of seeds that sank was recorded daily. After 35 days the sinking rate had plateaued, and a Tukey's multiple comparison test (α = 0.05) was used to test for differences. Only UVXPBR was not statistically different (p>0.05) from the unmarked control. Therefore, UVXPBR was selected as the medium to mark all seed due to the negligible impact on seed buoyancy. Thus, we showed that some methods of marking seed are undesirable as they may impact the buoyancy characteristics of seed floatation (e.g., see [26]). We acknowledge other factors such as seed surface roughness and morphology could impact dispersal but felt buoyancy was the most critical component.

### In situ non-riparian water dispersal

Lots of 500 seeds were marked with UVXPBR as above, which could be identified in the field with ultraviolet light. We created a dark environment hood with base dimensions 0.91×0.6 m defining the quadrat size for field recapture. The dark hood consists of a steel frame and tin siding with a plywood top that has camera-mounting hardware facing directly into the hood 91 cm above the ground. Four 14.5 cm battery operated black lights were then placed on all sides of the interior of the hood, causing the seeds to fluoresce. To ensure image uniformity, the camera was set to a shutter speed of 1/3 seconds and an aperture of 3.8.

The study was performed within Pandapas Pond Recreation Area in (37.282512N, −80.475025W) Jefferson National Forest (Giles County, VA) in an area already invaded with *M. vimineum* in from late January to late February of 2012 to represent a typical period when fully mature *M. vimineum* seed are present and likely dispersing in this region (Tekiela personal observations). A total of 10 transects were selected to maintain an oak (*Quercus* spp.), tulip-poplar (*Liriodendron tulipifera*), and American beech (*Fagus grandifolia*) overstory and litter composition. Transects were kept within 200 m of one another to ensure similar precipitation duration and intensity, and were oriented in the required direction to run parallel to the slope. Percentage leaf litter cover (0–100%) was visually estimated for each transect (min: 10%, max: 85%, average: 31±7%) and slope determined to range from 8.2 to 16.3° (average 9.6±0.7).

Marked seed lots were surface sown at the top of the transects in approximately 5 by 91 cm (width of dark box) strips running perpendicular to the slope in the middle of the first quadrat (Q1) of each transect. We determined that this density and spatial arrangement minimized seed clumping, which increases automated image analysis accuracy. Immediately after placing seeds onto soil surface we recorded a UV-image of Q1 and marked the corners of the dark hood with flags to assist in taking identical images after future rain events. We defined a line (D0 = the starting line) as the farthest point downslope that any one seed was found after initial imagery. Seed needed to travel beyond D0 to be considered dispersed. We recorded a second UV-image three days after the initial image was taken, but before any precipitation, to see if seed movement occurred during a rainless period and assess the initial recapture rate. We found that no seed moved within Q1 of any transect during this rainless period.

**Figure 1:**
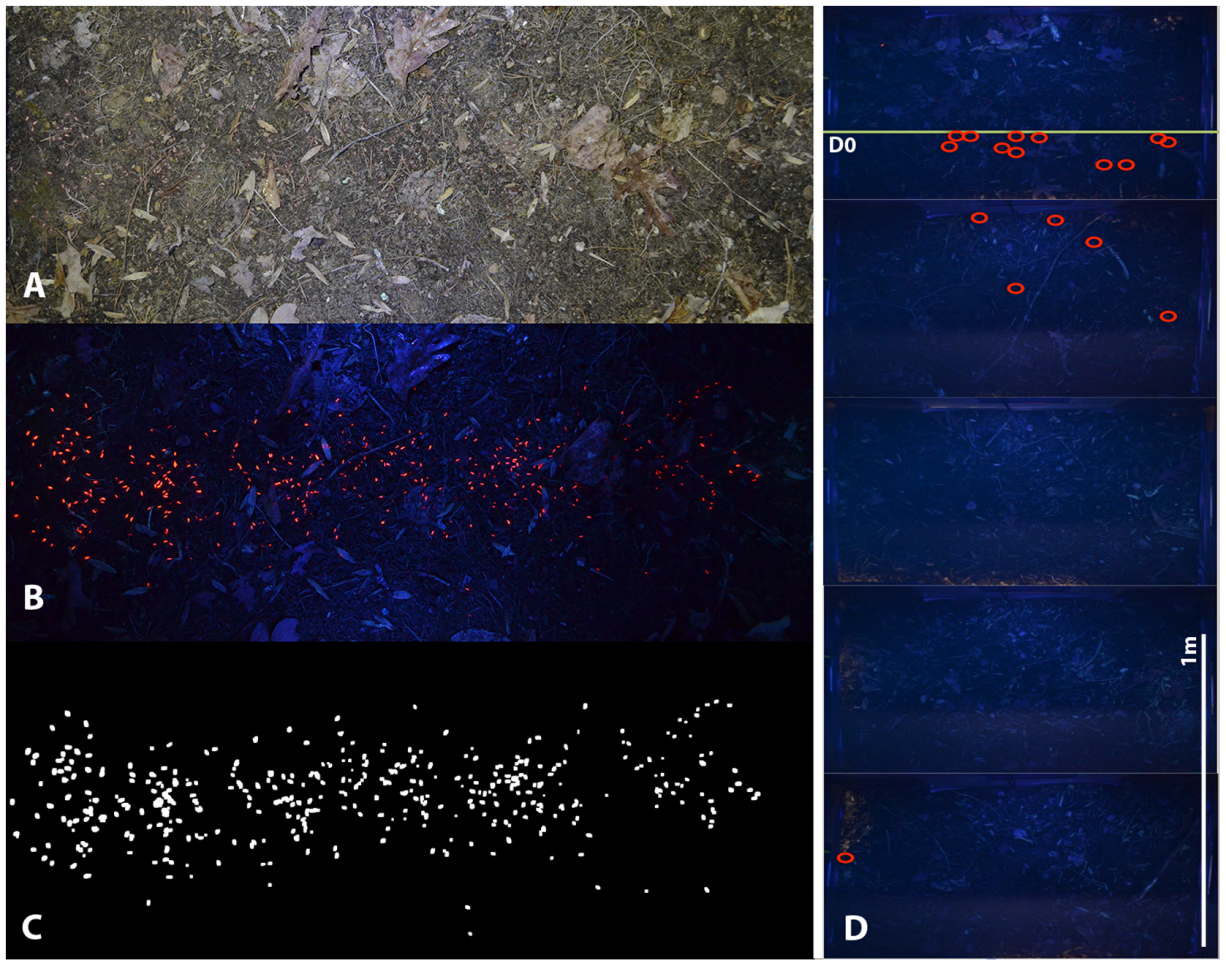
Transect Imagery. Example of Q1 with ambient light (A), UV assisted imagery (B), and its conversion to black and white (C) for automated analysis. The compiled UV images of an entire transect (D) with dispersed seed marked in red circles and 1 m scale bar.

We designed a macro in Adobe Photoshop CS5 that would allow for an accurate count of the total seeds in an image (Figure 1). Complete recapture (100%) even with the initial image is near impossible due to leaf litter and surface roughness. Therefore, this initial assessment was critical to identify the maximum effectiveness of our technique in seed recapture. We found the average rainless recapture rate was 62±12%. This was done for each transect after each rain event for the entire 5.5 m transect (Q1–Q6). Immediately following the first rain event, we scouted the soil surrounding the transects at night with UV lights to see if any movement occurred, and if so, make note of directionality. No dispersed seed were recorded at this time, as this was only to assist in directionality of dark box transects. Using the directionality from our scouting trip, we then took images of the entire transect using the method above. This process was repeated following four rain events after which the recapture rate was deemed too low to continue (<15%).

We summed all seeds within all quadrats of each transect to record how many seeds were recaptured after each rain event. Because consecutive rain events continually reduced the detection level of our recapture methodology, we adjusted seed counts for each rain event. This was done based on the probability of recapture for each rain event to estimate the total seed that moved but could not be recaptured. To do this we assumed that all seed remained within each transect. To test this assumption we scouted the area surrounding each transect for any seed that had moved beyond the boundaries of each transect, and found no seed. Therefore, we believe leaf litter, soil roughness, and soil particles “hide” a certain percentage of seed from top-down imagery. Thus, we assumed that any seed not captured by imagery still exists within each transect. Based on this, we adjusted the actual recorded seed to account for hidden seed. To adjust the seed quantities we took the counted seed and divided it by the detection probability to create our adjusted seed quantity.

**Figure 2:**
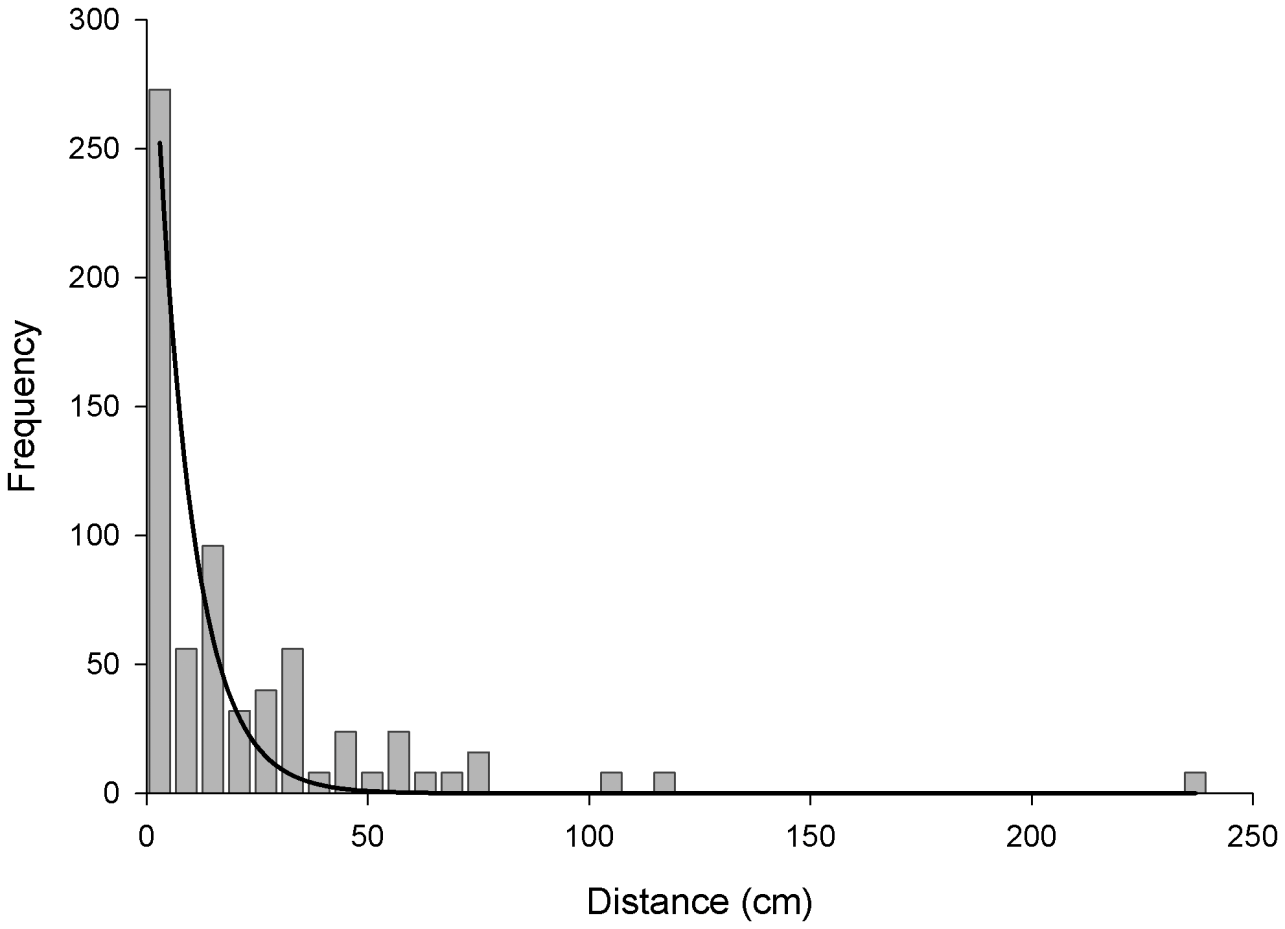
*M. vimineum* Non-riparian Water Dispersal Frequency Distribution. Frequency distribution of *M. vimineum* seed (excluding un-dispersed seed) from a total of 5000, dispersed by non-riparian water dispersal binned into 6 cm long segments after 4 rain events totaling in 52.3 mm of rain. Line represents best-fit logarithmic curve (y = 361.2^−0.12x^).

Any seeds that dispersed were binned into 3 cm segments for each rain event. Our precision is already limited because seed could have been initially placed anywhere within the 5 cm band so this binning did not reduce precision and made analysis possible. We then summed the amount of seed in each bin for each transect after rain event 4 to create a frequency distribution (Figure 2) that fits a logarithmic shape (*w_i_* = 0.6472) commonly seen in seed dispersal [27].

Parsing rain events and their individual effect on dispersal is impossible due to the inherent drawback of using a continuous series of rain events. We are unable to identify the starting point from which each dispersed seed originated. For example, seeds found to be dispersed following rain event 3 could have moved that distance exclusively from event 3, or they could have moved a portion of that during event 1 or 2. Therefore, we considered each new rain event to be an accumulation of prior rain events and the current event (i.e., event 2 = event 1+event 2). There were four rain events during the study: event 1 was 6.1 mm over 14.5 hours, event 2 was 11.2 mm over 15 hours, event 3 was 7.9 mm over 10 hours, and event 4 was 27.2 mm over 42 hours.

For some of our analyses, we used a metric we refer to as “total seed mobility” as our dispersal distance metric. The metric is simply each seed's travel distance summed over each transect. This metric is dependent on the total number of seed in each “population”, but because all transects started off with identical quantities of seed (500), comparisons can be made among the seed populations.

To test the effectiveness of our recapture method over varying leaf litter, a linear regression was used with recapture percentage of the entire transect as the dependent variable and litter cover as the independent variable. A linear regression was performed in JMP v9 (SAS Institute Inc.) with precipitation as the independent variable and total seed mobility as the response to test the effect of precipitation on seed dispersal. Blocking on each transect was used to control for variation in slope, leaf litter, and surface roughness and to meet requirements of data independence. A logarithmic transformation was used to meet the assumptions for parametric statistical analysis.

### Passive Seed Collection

To complement the manipulative study above, we conducted an observational study that began in December 2011 less than 2.5 km from the manipulative experiment above, also in Jefferson National Forest. This study was designed to passively quantify runoff and *M. vimineum* seed movement during rain events in an existing invasion. Three 2×2 m plots were installed on 32±0.2° slopes into both understory (>80% canopy cover, *M. vimineum* density 5,303±1,174 individuals m^−2^) and open canopy populations (<25% canopy cover, *M. vimineum* density 1,688±289 individuals m^−2^) of *M. vimineum* using steel borders installed 4 cm into the ground. We then installed a triangular flat funnel on the downslope edge of the plot. Therefore, all runoff from the 4 m^2^ area is collected. The funnel was connected to a bottle to collect any runoff from the defined plot. Precipitation volume and duration was collected using two rain buckets (one per site) connected to two data loggers recording data at 15-minute intervals. The study was performed from late December to early march, which included the time the *in situ* study was conducted. There were 11 precipitation events over a 3 month period.

Soil infiltration was determined at all sites using the Cornell Sprinkle Infiltrometer (Cornell University) and its original methodology [35]. All tests were performed on the same day to keep temporal soil moisture variation to a minimum. The rainfall rate was set to ∼2 cm sec^−1^, the collection ring was installed 15 cm into the ground, and runoff was collected until a plateau was reached as a volume of rainfall min^−1^. To test for site (passive & *in situ*) variation on maximum runoff rates, we performed an ANOVA (n = 10 α = 0.05).

## Results

**Figure 3:**
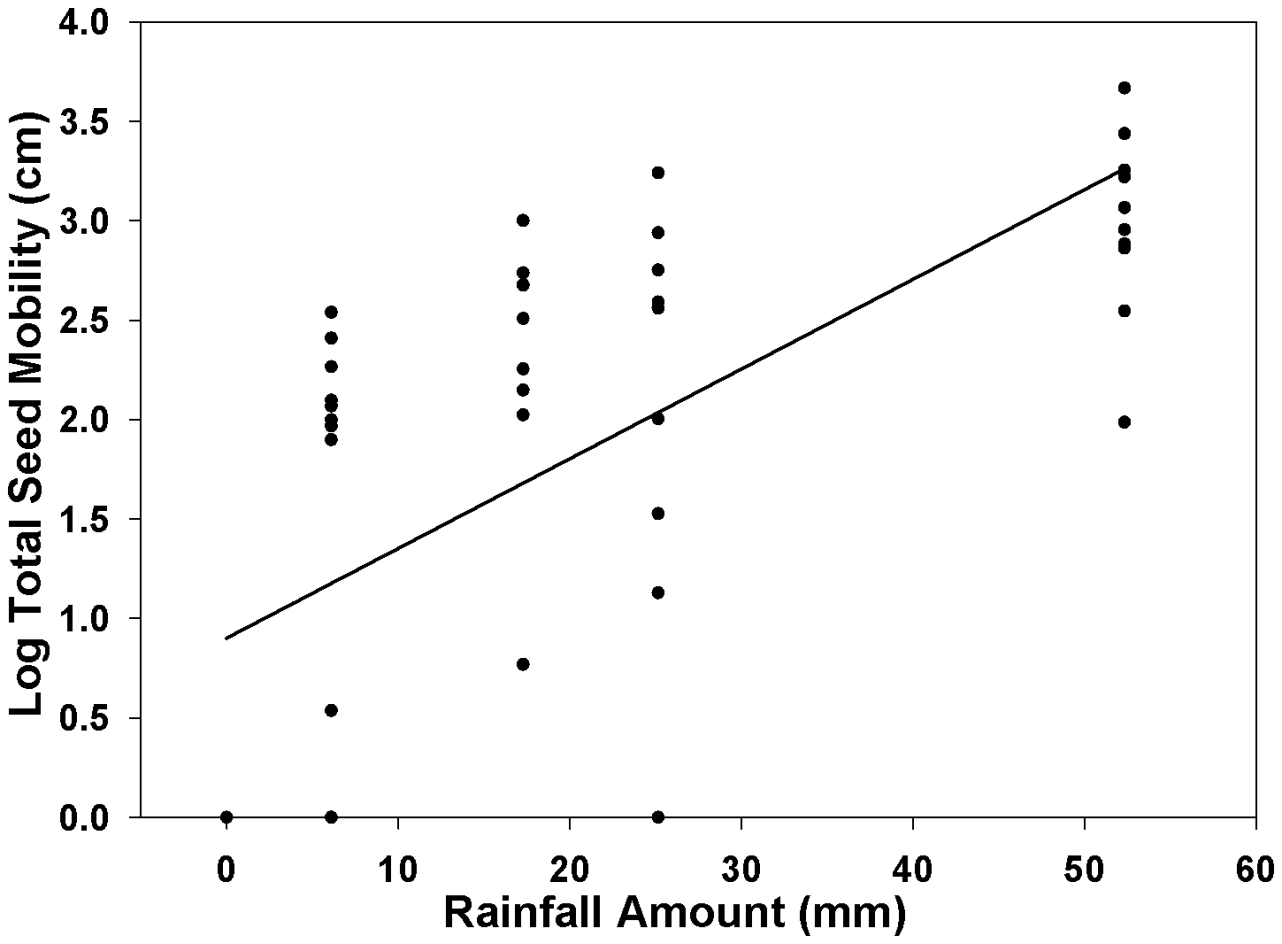
Precipitation Dispersal Response. Precipitation's influence on dispersal distance of *M. vimineum* by non-riparian water dispersal.

On average, the seed recapture rate immediately following surface sowing into Q1 for each transect was 73±13%, which is extremely high for a seed dispersal study [28]. Leaf litter was negatively correlated with seed recapture (p = 0.040). After the rainless period, detection rate decreased to 62±13%; 44±14% after rain event 1, 26±15% after event 2, 22±8% after event 3, and 12±4% after event 4. In contrast to the passive seed collection site, we recaptured non-riparian water dispersed *M. vimineum* seed in all 10 transects during each rain event, suggesting non-riparian water dispersal (i.e., overland flow) had occurred. In addition, no seed moved during the rainless period. Following the final rain event (cumulative precipitation of 52.3 mm), non-riparian water dispersal seed moved 21.1±3.6 cm on average, and the furthest detected seed traveled 2.4 m. Most seed (2.8%) dispersed to the 1–3 cm bin distance and the median of travel distance was 12–15 cm. The rate of movement per dispersed seed was 0.046±0.012 cm per 1 mm of rain. Total seed mobility was positively correlated with precipitation amount (p<0.0001, Figure 3).

Our passive seed collection study did not yield any overland water flow during any rain event in either the open or closed canopy environment. There was a statistical trend (p = 0.087) for the manipulative site (346±61 cm) compared to the passive site (170±66 cm).

## Discussion

Non-riparian water dispersal has previously been suggested in the literature to partially account for *M. vimineum* local population expansion, but has never been empirically tested. We demonstrated that non-riparian water dispersal does move *M. vimineum* seed in a hardwood forest understory, and likely contributes to local population expansion. Non-riparian water dispersal may partially explain why previous results of local population expansion are in conflict with field observations of rapid population expansion.

In our study, we wanted to ensure that seed movement (i.e., recaptured seed) was exclusively the result of non-riparian water dispersal. Therefore, we installed the plots at a time that would allow for a rainless period to occur, assuming that any additional vectors of seed movement would occur during this period (e.g., wind, animal). We could then perform data collection during a rainless period, and assess if any seed moved during this period. Using both our automated imagery analysis results and results from the scouting trip, we conclude there was no seed movement during this rainless period that could have been attributed to other vectors such as wind or animal. In addition, we only saw seed movement in a down-slope direction following rain events. Therefore, we conclude that the unidirectional downhill seed movement observed during our study was caused exclusively by overland water flow.

Though non-riparian water dispersal is rarely studied, this dispersal vector is the terrestrial analog to flood water and river corridor dispersal, as seed must remain buoyant and float in the “water column”. Many wetland species have evolved seed buoyancy to not only disperse their seed, but to also deposit them in optimal dry-down periods [34], [36], [37]. Though *M. vimineum* does not have any clear dispersal adaptations, it is able to remain buoyant for many weeks in water. Hydrochory is also a dispersal mechanism of riparian species, which depend on it to disperse buoyant seed and other propagules downstream from parent populations [38], [39], [40]. In fact, multiple studies have shown that hydrochory can act as a secondary dispersal vector for the invasive tree *Ailanthus altissima* [41], [42], which has an achene adapted to wind dispersal. In all cases, the ability to remain buoyant is important in hydrochory and this remains the case in non-riparian water dispersal. Seed buoyancy in *M. vimineum* may play a role during ephemeral sheetflow events.

*Microstegium vimineum* has been previously shown to disperse with water. Romanello [25] conducted a study in drainage ditches and showed that seed moved in channelized water, but this study only focused on the presence and absence of *M. vimineum* seed in different drainage pipes downstream from extant populations. Though this study showed hydrochory dispersed seed, it did not allow for dispersal distances to be quantified. Warren et al. [26] used a marking method similar to ours to track seed near commonly flooded areas. Using standard spray paint to mark the seed, they found plots near channelized water moved downslope from the parent patch. Though this method was similar to our own, the use of spray paint likely negatively impacted buoyancy dynamics, which likely altered the dispersal ability of marked seed.

Our primary focus was to determine the effect of precipitation amount on *M. vimineum* dispersal; however, other environmental variation (i.e., slope, leaf litter, soil roughness) may influence the dispersal distance of *M. vimineum* dispersal. For example, there is evidence to suggest that microsites with increased leaf litter and vegetation have more seeds associated with their seed banks [43], [44], possibly suggesting that increased leaf litter could act as a barrier to dispersal. Oswalt and Oswalt [13] suggested that leaf litter may inhibit realized dispersal on populations edges by limiting germination of *M. vimineum* via reduced seed to soil contact. However, Hull [45] and Schramm and Ehrenfeld [46] controlled propagule pressure in different leaf litter regimes and found leaf litter had no impact on germination. This suggests that leaf litter impacted dispersal and not germination in Oswalt and Oswalt [13]. Marshall and Buckley [47] further supported this idea by concluding that plots with relatively smooth surfaces had greater rates of population expansion. It also suggests that seed dispersal occurred after seed made soil contact, though they did not test this explicitly. Schramm and Ehrenfeld [20] also found that seedlings appeared downslope of parent patches two to four times more often than upslope. All of these conclusions support non-riparian water dispersal in *M. vimineum*, which we demonstrate empirically in *M. vimineum* for the first time.

Additionally, slope has been found to be an important factor influencing seed dispersal distance in other species. For example, Kaproth & McGraw [42] found *Ailanthus altissima* seed to move further with greater grades (48%–9% grade). Emmerson et al. [48] was similarly interested in dispersal of *Erodiophyllum elderi* seed heads by secondary dispersal vectors including sheet flow. Using fluorescent paint to visually recapture seed heads, the study found greater slope increased mean seed head dispersal distance. Future research should look at the interaction of soil roughness, leaf litter, and slope on the dispersal dynamics of *M. vimineum*.

In the context of *M. vimineum* population-level expansion, we demonstrated that non-riparian water acts as an effective dispersal vector. Currently, stem lodging is reported to only disperse seeds ∼0.5 m yr^−1^ [49]. Non-riparian water dispersal may be capable of expanding populations at over double that rate in downslope directions depending on the propagule pool size, site characteristics, and precipitation intensity and duration. Unexpectedly, we found that our passive precipitation collection system did not collect any overland flow or seed during a 4-month period. However, the soil infiltration rate at this site was greater. Emmerson et al. [48] found sites with clay soils dispersed seed heads further than sandy sites. Though not explicitly stated in the paper, variation in soil infiltration may have played a role in this result. This also is in line with our (and others) observations of *M. vimineum* often occurring in low spots in the landscape, areas likely to experience ephemeral water flow.

As some of our initial imagery results show, litter cover appears to negatively impact recapture efficacy. If this relationship holds throughout the entire experiment for the entire transect, then detection rate of long distance dispersal is also impacted by leaf litter which may impact perceived dispersal distance depending on leaf litter amount. It is because of this potential issue that we avoid using a mean dispersal distance, which may have been skewed by capture of few long distance events, and instead used total seed mobility. Regardless, this is a limitation to this recapture technique as is detection of long distance dispersal events in most if not all dispersal tracking methods [27].

This UV recapture method is successful at locating *M. vimineum* dispersed by non-riparian water dispersal. UV marking can be an effective method for tracking other propagules in various systems. Because this marking method has no impact on propagule buoyancy, it could be used in other hydrochorous systems (i.e., riparian dispersal, flood dispersal) that have been difficult to implement traditional trapping and tracking techniques in. The potential sampling bias of trapping can be overcome by scouting the entire potential dispersal area, such as was done in this study. Also, UV recapture does not require the removal of seed or any other intrusive techniques to relocate seed like traps.

## Conclusion

Non-riparian water dispersal is a newly studied mechanism by which *M. vimineum* populations expand at the local scale. Though we fully acknowledge the limitations of using only a single site with one year of data, we are confident that we have demonstrated non-riparian water dispersal in *M. vimineum*. Non-riparian water dispersal may partially explain many of the anecdotal accounts of *M. vimineum* expanding at rates far greater than current literature reports. Based on buoyancy tests and our dispersal study, our data suggest that forests with periodic overland flow may also be at a greater risk of *M. vimineum* population expansion. Further studies aimed at understanding how *M. vimineum* seed is moved by non-riparian water at a larger geographic scale will increase our understanding of this unique component of hydrochory, and could lead to a better understanding of propagule dispersal in general.
